# *TP53* Combined Phenotype Score Is Associated with the Clinical Outcome of *TP53*-Mutated Myelodysplastic Syndromes

**DOI:** 10.3390/cancers13215502

**Published:** 2021-11-02

**Authors:** Mariko Yabe, Aidana Z. Omarbekova, Meier Hsu, Hannah May, Maria E. Arcila, Ying Liu, Ahmet Dogan, Andrew M. Brunner, Valentina Nardi, Robert P. Hasserjian, Virginia M. Klimek

**Affiliations:** 1 Hematopathology Service, Department of Pathology, Memorial Sloan Kettering Cancer Center, New York, NY 10065, USA; liuy6@mskcc.org (Y.L.); dogana@mskcc.org (A.D.); 2 Leukemia Service, Department of Medicine, Memorial Sloan Kettering Cancer Center, New York, NY 10065, USA; Aidana.Omarbekova@uth.tmc.edu (A.Z.O.); mayh@hss.edu (H.M.); virginiaklimekmd@gmail.com (V.M.K.); 3 Department of Epidemiology and Biostatistics, Memorial Sloan Kettering Cancer Center, New York, NY 10065, USA; hsum1@mskcc.org; 4 Diagnostic Molecular Pathology Service, Department of Pathology, Memorial Sloan Kettering Cancer Center, New York, NY 10065, USA; arcilam@mskcc.org; 5 Department of Hematology/Oncology, Massachusetts General Hospital, Harvard University, Boston, MA 02115, USA; abrunner@partners.org; 6 Department of Pathology, Massachusetts General Hospital, Harvard University, Boston, MA 02115, USA; VNARDI@PARTNERS.ORG (V.N.); RHASSERJIAN@mgh.harvard.edu (R.P.H.)

**Keywords:** myelodysplastic syndromes, *TP53*, prognosis, overall survival, PHANTM combined phenotype score

## Abstract

**Simple Summary:**

*TP53* is the most frequently mutated genes in cancer, and mutations of *TP53* are observed in 5–10% of patients in myelodysplastic syndrome (MDS). In patients with MDS, *TP53* mutations are associated with adverse outcomes; however, there is still significant heterogeneity in these disease courses. We performed retrospective review of 107 patients with untreated *TP53*-mutated MDS, and identified that the functional impact of *TP53* mutations, represented by phenotypic annotation of *TP53* mutations (PHANTM) combined phenotype score is associated with prognosis. In patients with *TP53*-mutated MDS, we found that a higher PHANTM combined phenotype score is associated with poorer clinical outcome, and this has independent influence on prognosis accounting for IPSS-R and other risk variables. Our findings suggest that *TP53*-mutated MDS is heterogeneous and not all *TP53* mutations harbor the same impact on prognosis. The PHANTM combined score adds to prognostic precision in MDS beyond previously reported *TP53* allelic state.

**Abstract:**

Mutations of *TP53* are observed in 5–10% of patients in myelodysplastic syndrome (MDS) and are associated with adverse outcomes. Previous studies indicate that the *TP53* allelic state and variant allele frequency of *TP53* mutation impact patient outcomes, but there is significant heterogeneity within this MDS subgroup. We performed retrospective review of clinicopathologic and genomic information of 107 patients with *TP53*-mutated MDS. We assessed each mutation according to the phenotypic annotation of *TP53* mutations (PHANTM) and analyzed the associations between predicted *TP53* mutant function, represented by the PHANTM combined phenotype score, and overall survival (OS) using the log rank test and Cox regression. Our results indicated that patients with PHANTM combined phenotype score above the median (>1) had significantly shorter OS compared to those with scores below the median (median OS: 10.59 and 16.51 months, respectively, *p* = 0.025). This relationship remained significant in multivariable analysis (HR (95%CI): 1.62 (1.01–2.58), *p* = 0.044) and identified to have an independent prognostic influence, accounting for known risk such as IPSS-R and other standard risk variables. Our results suggest that the functional information of *TP53* mutations, represented by PHANTM combined phenotype score, are associated with the clinical outcome of patients with *TP53*-mutated MDS.

## 1. Introduction

Myelodysplastic syndrome (MDS) is a heterogeneous group of myeloid disorders characterized by ineffective hematopoiesis leading to cytopenias and risk of transformation to acute myeloid leukemia (AML) [1]. *TP53*, a tumor suppressor gene, is the most frequently mutated gene in cancer, and mutations of *TP53* are observed in 5–10% of patients with MDS [1,2,3,4]. In patients with MDS, *TP53* mutations are associated with high-risk disease, rapid transformation to AML, resistance to conventional therapies, and shorter survival [5,6,7,8,9,10].

While *TP53* mutations are known to be associated with complex karyotypes and generally predict poor prognosis in myeloid neoplasms, associations between outcome and the predicted functional deficit of specific *TP53* mutations have not been well characterized [5]. A tumor suppressor gene is a gene that regulates cell division and replication, and a loss or reduction in its function caused by gene deletion, truncation, or alteration in promoter lesions results in uncontrollable cell growth and leads to oncogenesis [11,12]. However, unlike most tumor suppressor genes, the most common genetic alterations in *TP53* are missense mutations which can happen throughout the gene [3,13]. It has previously been argued that loss of p53 function is the critical determinant in cancer, yet the preponderance of missense mutations relative to truncation mutations also argues that full-length mutant p53 actively promotes tumor development [14,15]. Mutant p53 protein is often abundantly expressed in cancers and specific allelic variants exhibit dominant-negative or gain-of-function activities in experimental models [16,17,18,19,20,21]. Experimental models also show that over 80% of full-length *TP53* DNA-binding domain missense mutants that displays loss-of-function also display dominant-negative activity, suggesting that the ability of mutant p53 to interfere with wild type p53 is critical during tumorigenesis [15]. To determine the function of each missense or nonsense *TP53* mutations, Giacomelli et al. created a comprehensive library of p53 mutants and evaluated the function of these alleles in the presence or absence of endogenous p53 [15]. From these data, they developed a classifier which provides functional classification for any missense and nonsense variant of *TP53* (phenotypic annotation of TP53 mutations (PHANTM); http://mutantp53.broadinstitute.org, accessed on 4 February 2020) [15].

In this study, we estimated p53 mutant function, represented by the PHANTM combined phenotype score, and determined the impact of this score on the overall survival (OS) of patients with *TP53*-mutated MDS.

## 2. Materials and Methods

### 2.1. Study Group

We searched the medical records of Memorial Sloan Kettering Cancer Center and Massachusetts General Hospital, Harvard Medical School, for cases diagnosed with MDS with *TP53* mutation between January 2007 and December 2019. Search was focused on identifying the patients with no prior disease-modifying therapy for MDS, i.e., no prior cytotoxic therapy for MDS with or without a history of transfusion and growth factor administration. The diagnosis of MDS was based on criteria specified in the World Health Organization classification [1]. We performed a retrospective review of the medical records. As part of the chart review, we collected demographic information, history of any prior cytotoxic therapies, the clinical presentation, findings on physical examination, underlying diseases, laboratory data, therapy, and clinical follow-up. Prognostic risk was calculated using the revised International Prognostic Scoring System (IPSS-R) [22]. These data were collected according to protocols approved by the institutional review boards of all institutions in accordance with the Declaration of Helsinki.

### 2.2. Conventional Cytogenetics and Fluorescence In Situ Hybridization Analysis

Conventional karyotyping was performed on bone marrow aspirate and reported by using the International System for Human Cytogenetic Nomenclature (ISCN) 2013 [23]. Fluorescence in situ hybridization (FISH) analysis was performed with BM aspirate on a subset of cases to assess for loss of 17p using LSI *TP53*/CEP17 FISH Prove Kit (Abbott Molecular/Vysis, Des Plaines, IL, USA). A total of 300 interphases were analyzed. The cutoff established in our laboratory was 3% for *TP53* (17p13.1) deletion.

### 2.3. Next-Generation Sequencing

Next-generation sequencing was performed using different platforms that evaluated similarly compiled 30 myeloid gene panels in addition to *TP53*. Many of these cases were evaluated by a next-generation sequencing-based custom-designed assay using the Illumina MiSeq platform. *TP53* mutations were characterized by variant type and exon location, and variant allele frequency (VAF). The number of other co-mutated genes was also recorded. The limit of detection for variant calling was 2%.

### 2.4. TP53 Mutation Phenotype Score (PHANTM Combined Phenotype Score)

We applied phenotypic annotation of TP53 mutations (PHANTM); http://mutantp53.broadinstitute.org (accessed on 4 February 2020) for detected missense and nonsense *TP53* mutations, and PHANTM combined phenotype score was calculated for each patient. This supports functional classification for missense and nonsense variants in the gene *TP53*; therefore, for the cases with frameshift mutation, in-frame deletion, or splice site mutation, we applied the score of 1 based on literatures that shows pathogenic features of these gene alterations [24,25].

### 2.5. Statistical Analysis

The primary endpoint of this study was overall survival (OS) time, defined as the time from the date of MDS diagnosis until the date of death or last follow-up. Only patients with *TP53* mutations were included in this study; therefore, we account for the interval from diagnosis of MDS until *TP53* sequencing, when patients enter the risk set, using left-truncation [26]. Median OS was estimated using the Kaplan–Meier method and compared between groups using the log rank test. Continuous variables were assessed using Cox regression. We explored combined phenotype score as both a categorical variable dichotomized at the median and as a continuous variable. Combined phenotype score was evaluated for independent association with OS in multivariable Cox regression models controlling for clinically and statistically significant factors. Combined phenotype score was also evaluated for association with important clinical factors using Pearson’s Chi-squared test or the Wilcoxon rank sum test. AML transformation and transplant were analyzed for association with OS as time-dependent covariates in Cox regression models. A *p* value of <0.05 was considered statistically significant. All statistical analyses were performed in R version 3.6.1 (R Foundation for Statistical Computing, Vienna, Austria).

## 3. Results

### 3.1. Patient Characteristics

We identified 107 patients with *TP53*-mutated MDS with no prior disease modifying therapy. Patient characteristics are summarized in Table 1.

### 3.2. TP53 Mutations, Other Co-Mutations, and Cytogenetic Abnormalities

#### 3.2.1. TP53 Mutations

**Figure 1:**
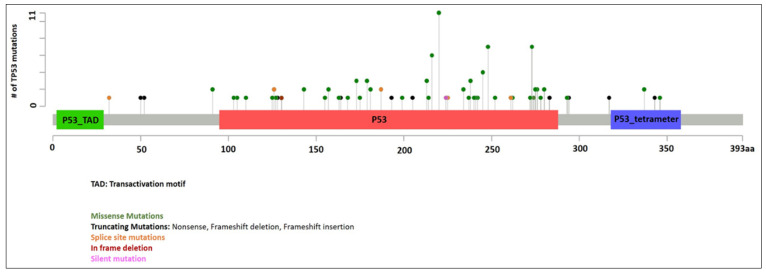
*TP53* mutations identified in our cohort.

Information of *TP53* mutations is summarized in Figure 1. A total of 128 mutations were identified in 107 patients with median VAF of 0.29 (range 0.02 to 1.00). Twenty-one patients had two *TP53* mutations. VAF was not available in three patients (all with one *TP53* mutation). In patients with two mutations, the *TP53* mutations with the higher VAF were designated as the primary *TP53* mutations in this study for evaluation purpose. The median VAF of the primary *TP53* mutations were 0.34 (range 0.02 to 1.00, *n* = 104). Missense mutations were the most common (86 patients, 80%), followed by splice site mutations (seven patients, 6.5%), frameshift mutations (six patients, 5.6%), nonsense mutations (five patients, 4.7%), and in-frame deletions (three patients, 2.8%). The median VAF of secondary *TP53* mutations were 0.22 (range 0.04 to 0.44, *n* = 21). Missense mutations were again the most common (12 patients, 57%), followed by nonsense mutations (four patients, 19%), splice site mutations (four patients, 19%), and frameshift mutations (one patient, 4.8%).

#### 3.2.2. Other Co-Mutations

**Figure 2:**
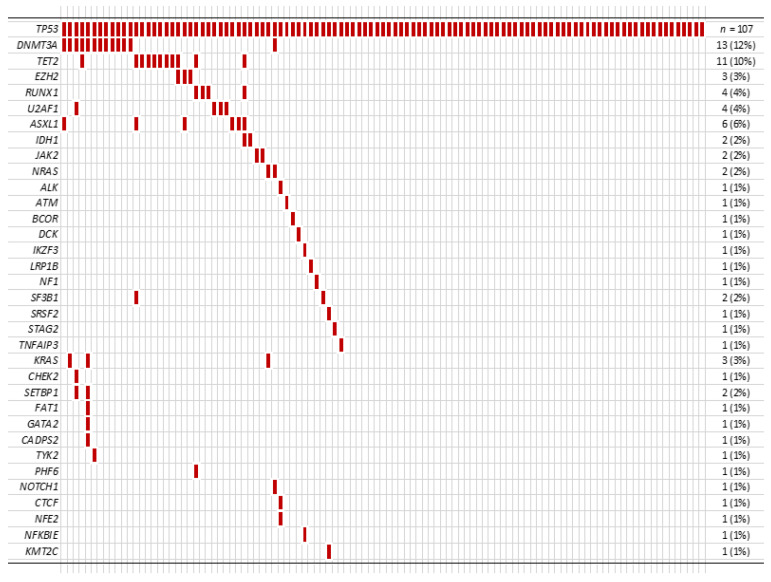
Other co-mutations identified in our cohort.

Among 107 patients, 47 patients (44%) had additional mutation(s) in addition to the *TP53* mutation (Figure 2). In these patients with co-mutation(s), the median number of additional mutation(s) was 1 (range 1 to 6), and *DNMT3A* was the most frequent co-mutation (13/107, 12%) followed by *TET2* (11/107, 10%).

#### 3.2.3. Cytogenetic Abnormalities

Cytogenetic data were available for 104 patients, and 33 patients showed loss of 17p by conventional karyotype and/or FISH. Fifty patients had one *TP53* mutation with no loss of 17p, 21 patients had two *TP53* mutations with no loss of 17p, and 33 patients had one *TP53* mutation with loss of 17p.

### 3.3. PHANTM Combined Phenotype Score

PHANTM combined phenotype score was calculated from the primary mutation in each patient. It ranged from −0.559 to 1.778 (median 1.00) (Table S1). PHANTM combined phenotype score, when analyzed as either continuous or as binary variable dichotomized at the median, was not significantly associated with the number of *TP53* mutations or the presence of other co-mutations (Table 2).

### 3.4. Therapy

During the follow up, 39 patients (36.4%) showed transformation to AML. Among these 39 patients who progressed to AML, 15 patients (38.5%) received stem cell transplant. Among 68 patients who did not progress to AML, 23 patients (33.8%) received stem cell transplant.

### 3.5. Outcome

**Figure 3:**
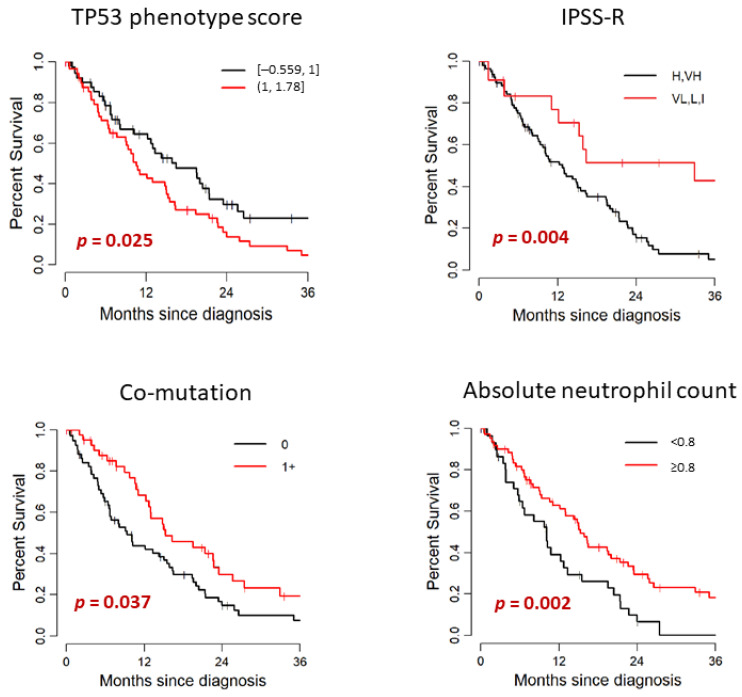
Univariate analysis for association with OS.

Patient’s outcome and its association with clinical and mutational characteristics are summarized in Table 3 and Figure 3. In the study cohort of 107 patients, 79 died. The median follow-up after diagnosis was 12.8 months (range: 0–50.5) among survivors. Median OS months (95% CI) was 14.84 (11.09–19.51). Median time from diagnosis to *TP53* testing was 0.2 months (range: 0–40.7). *TP53* testing was performed within 3 months of diagnosis for 97 patients (91%) and after 3 months for 10 patients (9%); the median time to *TP53* testing for this subset was 9 months (range: 3.6–40.7). There was no significant difference in OS between patients with de novo MDS and therapy-related MDS (median OS: 15.07 and 10.59 months, respectively. *p* = 0.674). When we compared the WHO categories stratified by blast percentage, there was no significant difference in OS between MDS-EB-1/2 and MDS without excess blasts (median OS: 15.56 and 11.09 months, respectively. *p* = 0.806). The OS of lower risk IPSS-R groups (VL, L, I) was significantly longer than the IPSS-R higher risk groups (VH, H) (median OS: 32.93 and 12.76 months, respectively, *p* = 0.004). When we stratified by IPSS-R cytogenetic risk groups, the OS of lower risk groups (good, intermediate) showed tendency to have longer survival than the higher risk groups (poor, very poor); however, this finding was not statistically significant in our cohort (median OS: 23.52 and 13.03 months, respectively, *p* = 0.14). Cytopenia was also associated with poorer prognosis. This finding was statistically significant for the hemoglobin level (<10 vs. ≥10 g/dL, *p* = 0.036) and absolute neutrophil count (<0.8 vs. ≥0.8 × 10^9^/L, *p* = 0.002) in our cohort. Higher hemoglobin level and higher absolute neutrophil count were significantly associated with better outcome.

When we analyzed the genetic data, median OS was significantly shorter for patients with PHANTM combined phenotype score above the median (>1) than that of the patients with scores below the median (≤1) (median OS: 10.59 and 16.51 months, respectively, *p* = 0.025). When analyzed as a continuous variable, higher PHANTM combined phenotype score was significantly associated with increased risk of death (HR (95%CI):1.77 (1.04–3.01), *p* = 0.035). Sixteen patients had PHANTM combined phenotype score of 1, and all of these patients had either frameshift mutation (six patients), in-frame deletion (three patients), or splice site mutation (seven patients). Therefore, we also evaluated the prognostic influence of PHANTM combined phenotype score by univariate analysis among these 3 groups; <1, 1 and >1. We observed the consistent findings (*p* = 0.027); however, due to the small number of patients in the middle group, the confidence interval of the median OS estimate was wide (Table S2). Patients with two *TP53* mutations showed a tendency to have shorter survival than patients with one *TP53* mutation (median OS: 8.16 versus 14.31 months, respectively, *p* = 0.094). *TP53* VAF did not show statistically significant impact on prognosis both by either continuous or categorical analysis (<0.2 vs. 0.2–0.5 vs. >0.5). Locus of *TP53* mutation and *TP53* mutation type (missense vs. other) did not significantly differentiate patients on survival. The median OS was significantly longer for patients with 1+ co-mutations than that of the patients with no co-mutation (median OS: 15.33 and 9.38 months, respectively, *p* = 0.037). Patients who had one *TP53* mutation and loss of chromosome 17p had shorter OS than that of the patients with one *TP53* mutation with no loss of chromosome 17p (median OS: 12.76 and 16.38 months, respectively), and the patients with two *TP53* mutations had the shortest OS among these three groups (OS: 8.16 months). However, these findings did not reach statistical significance (*p* = 0.07).

We did not see the statistical difference of prognosis between patients who received or not received allogeneic transplant (HR (95%CI): 0.82 (0.30–2.26), *p* = 0.71), or between patients who transformed or not transformed to AML (HR (95%CI): 0.58 (0.17–1.97), *p* = 0.39) in our cohort.

The results of multivariable analysis for association with OS are summarized in Table 4. PHANTM combined phenotype score as a categorical variable, dichotomized at the median (over 1 vs. equal or less than 1), was found to be an independent prognostic factor in the patients with *TP53* mutated MDS (HR (95%CI): 1.62 (1.01–2.58, *p* = 0.044). Among other factors analyzed, IPSS-R showed independent prognostic significance (HR (95%CI): 0.42 (0.19–0.91), *p* = 0.028 for low vs. high risk groups). Having two *TP53* mutations was also independently associated with increased risk of death (HR (95%CI): 1.84 (1.04–3.25), *p* = 0.037). Finding was not statistically significant when analysis was performed with PHANTM combined phenotype score as a continuous variable (HR (95%CI): 1.53 (0.87–2.71, *p* = 0.14).

## 4. Discussion

Mutations in *TP53* are observed in 5–10% of patients with MDS and are associated with adverse outcomes [8,9,10,27]. Preclinical models suggest that distinct mutation types in different *TP53* mutation types in different *TP53* gene domains may lead to different impacts on protein functionality [28,29,30,31]. Whether these functional differences translate into distinct clinical features and outcomes and how the type, number, and size of *TP53* mutations influence the prognosis of patients with *TP53*-mutated MDS remains unclear. We applied the *TP53* phenotypic scoring system created by Giacomelli et al. to annotate the P53 mutant function and analyzed its association with outcome. We identified that the median OS was significantly shorter for patients with PHANTM combined phenotype score above the median (>1) than that of the patients with scores below the median. When analyzed as a continuous variable, higher PHANTM combined phenotype score was associated with increased risk of death. By multivariable analysis, PHANTM combined phenotype score as a categorical variable, dichotomized at the median (over 1 vs. equal or less than 1), was found to be an independent prognostic factor in the patients with *TP53* mutated MDS, along with other factors analyzed, including IPSS-R and having two *TP53* mutations. Our data suggest that the functional information of *TP53* mutations, represented by PHANTM combined phenotype score independently influence the clinical outcome in *TP53*-mutated myelodysplastic syndromes.

We applied the score of 1 for the cases with frameshift mutation, in-frame deletion, or splice site mutation, based on literatures that shows pathogenic features of these gene alterations [24,25]. In our cohort, 16 patients had PHANTM combined phenotype score of 1, and all of these patients had either frameshift mutation (six patients), in-frame deletion (three patients), or splice site mutation (seven patients). Therefore, we also evaluated the prognostic influence of PHANTM combined phenotype score by univariate analysis among these three groups: <1, 1, and >1. We observed the consistent findings (*p* = 0.027); however, due to the small number of patients in the middle group, the confidence interval of the median OS estimate was wide (Table S2). Analysis with larger cohort to obtain stable estimate is needed to confirm this finding in the future.

Detailed mechanisms of which factors of *TP53* mutation have the most influence on prognosis are not well defined in patients with *TP53*-mutated MDS, including the types of *TP53* mutation (missense, nonsense, or other), the number of *TP53* mutations, or VAF of the *TP53* mutation. One previous study showed that the types of *TP53* mutation (missense, nonsense, or other) or location of *TP53* mutations did not show significant association with OS, with only a trend to worse outcomes in patients with mutations not involving DNA-binding domain [32]. Another study performed in the post-transplant setting showed that patients with *TP53* truncating mutations (frameshift, nonsense, or splice site) had poor survival compared to those with missense only or missense plus truncating mutations [7]. Previous studies also showed that there was no correlation between the number of *TP53* mutations and OS [7,32]. In our study, based on the results of univariate analysis and previously published data, we chose IPSS-R, number of *TP53* mutations, loss of 17p, and number of co-mutations as the variables for multivariate analysis along with PHANTM combines phenotype score [22,33]. Patients with one *TP53* mutation had longer OS than patients with 2 *TP53* mutations, and the finding was statistically significant only by multivariate analysis. Our study showed no significant correlation between the type or location of *TP53* mutations and OS.

A recent study by Bernard et al. reported the importance of the allelic status of *TP53* [33]. They reported that the multi-hit *TP53* state (more than one gene mutation, mutation and deletion, mutation and copy-neutral loss of heterozygosity) in MDS underlies established associations with genome instability, treatment resistance, disease progression and dismal outcomes, indicating that consideration of *TP53* allelic state is critical for diagnostic and prognostic precision in MDS [33]. The results of our study support this finding, since the patients with one *TP53* mutation with no loss of chromosome 17p had longer OS than that of the patients with *TP53* mutation and loss of chromosome 17p (median OS: 12.76 and 16.38 months, respectively), and the patients with 2 *TP53* mutations had the shortest OS among these three groups (OS: 8.16 months). However, these findings did not reach statistical significance in our limited cohort of 107 patients (*p* = 0.07). This may reflect the relatively small size of our cohort or the inability to detect copy-neutral LOH in our study, which represented 20% of the *TP53* multi-hit cases in the Bernard et al. study [33]. Although the prognostic significance of the *TP53* VAF reported in several studies could be explained by a correlation between high VAF and multi-hit *TP53* status, some studies, including ours, did not find *TP53* VAF to correlate with outcome [7,10,32,34,35]. The VAF is influenced by sample quality (hemodilution) and the presence of normal background hematopoietic cells, thus it may not accurately reflect the single versus multi-hit status of the *TP53* gene.

Our study also showed that co-mutation(s) of other oncogenic genes (other driver mutations), IPSS-R and cytopenias are significantly associated with prognosis. It has been previously reported that the total number of co-mutations differ among the *TP53* allelic status [33]. Patients with monoallelic *TP53* mutations frequently have several co-mutations, while patients with multi-hit *TP53* state have fewer co-mutations [33]. Our study showed that the median OS was significantly longer for patients with 1+ co-mutations than that of the patients with no co-mutation (median OS: 15.33 and 9.38 months, respectively, *p* = 0.037). This is likely reflecting the comparison of OS between patients with monoallelic *TP53* mutations and patients with multi-hit *TP53* state. Blast percentage and preceding cytotoxic therapy have been reported to be associated with prognosis in *TP53*-mutated MDS; however, we did not see any difference in prognosis between MDS with excess blasts and other MDS, or between de novo and therapy-related disease in our cohort [32,36].

## 5. Conclusions

We found that the functional status of *TP53* mutations, represented by PHANTM combined phenotype score, is independently associated with the clinical outcome in patients with *TP53*-mutated MDS. In patients with *TP53*-mutated MDS, higher PHANTM combined phenotype score is associated with poorer clinical outcome. Our findings suggest that *TP53*-mutated MDS is heterogeneous and that not all *TP53* mutations harbor the same impact on prognosis. Use of the PHANTM combined score may help improve prognostic precision in MDS in addition to the previously reported *TP53* allelic state.

## Figures and Tables

**Table 1:** Clinical characteristics of patients (*n* = 107) with MDS with *TP53* mutation.

Clinical Characteristics	*n* (%)
**Age (Median), Year**	
Median	73
Range	24–91
**Gender**	
Male	72 (67)
Female	35 (33)
**MDS Type**	
de novo MDS	57 (53)
Therapy-related MDS	50 (47)
**Time from MDS Diagnosis to *TP53* Testing, Month**	
Median	0.3
Range	0–41.2
**WHO subtypes (including Therapy-Related MDS)**	
MDS-SLD	1 (0.9)
MDS-MLD	56 (52)
MDS-RS-MLD	4 (3.7)
MDS with isolated del5q	2 (1.9)
MDS-EB-1	26 (24)
MDS-EB-2	18 (17)
**IPSS-R**	
Very High	62 (60)
High	23 (22)
Intermediate	11 (11)
Low	5 (4.8)
Very Low	3 (2.9)
Unknown (due to lack of cytogenetic data)	3 (NA)
**IPSS-R Cytogenetic Risk Groups**	
Very good	0 (0)
Good	11 (11)
Intermediate	2 (1.9)
Poor	12 (12)
Very poor	79 (76)
Unknown (due to lack of cytogenetic data)	3 (NA)
**CBC**	
**Hemoglobin, g/dL**	
Median	8.7
Range	4.0–14.2
**Platelets, ×10^9^/L**	
Median	60
Range	1–422
**ANC, ×10^9^/L**	
Median	1.14
Range	0.01–8.63
**Blast %, Bone Marrow Differential Count**	
Median	6
Range	0–18

**Table 2:** PHANTM combined phenotype score (*n* = 107).

Variables	Range	Median	
PHANTM combined phenotype score	−0.559 to 1.78	1.00	
	**Number of *TP53* mutations**		
	1 (*n* = 86)	2 (*n* = 21)	*p* value *
PHANTM combined phenotype score (Median, (IQR))	1.00 (0.81, 1.22)	1.07 (0.99, 1.31)	0.3
PHANTM combined phenotype score			
−0.559 to 1	45 (52%)	9 (43%)	0.4
1< to 1.78	41 (48%)	12 (57%)	
	**Number of co-mutations**		
	0 (*n* = 60)	1+ (*n* = 47)	*p* value *
PHANTM combined phenotype score (Median, (IQR))	1.00 (0.90, 1.22)	1.04 (0.79, 1.33)	>0.9
PHANTM combined phenotype score			
−0.559 to 1	31 (52%)	23 (49%)	0.8
1< to 1.78	29 (48%)	24 (51%)	

*: Wilcoxon rank sum test; Pearson’s Chi-square test.

**Table 3:** Association of clinical and mutational characteristics with prognosis.

Clinical and Mutational Characteristics	*n*	Median Survival (Months)	95% CI *	*p* Value
Overall survival, all patients	107	14.84	11.09–19.51	NA
Diagnosis				
De novo MDS	57	15.07	10.79–23.49	0.674
Therapy-related MDS	50	10.59	8.06–16.51	
MDS-excess blasts	44	15.56	10.59–21.35	0.806
MDS-other	63	11.09	6.58–15.33	
IPSS-R				
Very High, High	85	12.76	9.77–16.32	**0.004**
Intermediate, Low, Very Low	19	32.93	15.33–NA	
IPSS-R Cytogenetic risk groups				
Good, Intermediate	13	23.52	12.76–NA	0.140
Poor, Very Poor	91	13.03	10.1–16.38	
Hemoglobin level (g/dL)				
<10	74	12.17	7.66–16.38	**0.036**
≥10	32	15.92	11.09–35.1	
Platelet count (×10^9^/L)				
<100	80	13.03	10.1–19.44	0.149
≥100	26	13.32	9.77–NA	
Absolute neutrophil count (×10^9^/L)				
<0.8	39	10.10	5.89–15.56	**0.002**
≥0.8	67	15.33	12.96–22.6	
Number of *TP53* mutations				
1	86	14.31	10.59–20	0.094
2	21	8.16	5.89 -19.61	
*TP53* VAF				
<0.2	31	9.77	6.74–20.39	0.858
0.2–0.5	42	13.03	10.1–21.41	
>0.5	31	16.32	10.23–23.49	
Number of co-mutations				
0	60	9.38	6.71–15.92	**0.037**
≥1	47	15.33	12.76–25.66	
*TP53* mutation and 17p loss				
1 *TP53* mutation and no loss of 17p	50	16.38	12.17–25.66	0.07
1 *TP53* mutation and loss of 17p	33	12.76	9.38–21.35	
2 *TP53* mutations	21	8.16	5.89–19.61	
PHANTM combined phenotype score				
−0.559 to 1	54	16.51	12.27–23.52	**0.025**
>1 to 1.778	53	10.59	8.98–15.33	
**Analysis as a continuous variable**		**HR**	95% CI *	***p* value**
PHANTM phenotype score	107	1.77	1.04–3.01	**0.035**
*TP53* VAF	104	0.99	0.91–1.08	0.817

* Confidence Interval; **BOLD** *p* value <0.05.

**Table 4:** Multivariate analysis for association with OS.

Variables	HR *	95% CI ^#^	*p* Value
**IPSS-R**			
Very High, High	─	─	
Intermediate, Low, Very Low	0.42	0.19–0.91	**0.028**
**Number of *TP53* mutations**			
1	─	─	
2	1.84	1.04–3.25	**0.037**
**Number of co-mutations**			
0	─	─	
1+	0.70	0.43–1.14	0.2
**Loss of 17p**			
No	─	─	
Yes	0.97	0.59–1.58	0.9
**PHANTM combined phenotype score (Binary analysis)**			
−0.559 to 1	─	─	
>1 to 1.778	1.62	1.01–2.58	**0.044**
	HR *	**95% CI ^#^**	***p* value**
**IPSS-R**			
Very High, High	─	─	
Intermediate, Low, Very Low	0.40	0.18–0.88	**0.023**
**Number of *TP53* mutations**			
1	─	─	
2	1.80	1.01–3.20	**0.045**
**Number of co-mutations**			
0	─	─	
1+	0.74	0.45–1.21	0.2
**Loss of 17p**			
No	─	─	
Yes	0.97	0.59–1.59	>0.9
**PHANTM combined phenotype score (continuous analysis)**	1.53	0.87–2.71	0.14

* Hazard Ratio; # Confidence Interval; **BOLD** *p* value <0.05.

## Data Availability

The data presented in this study are available in Supplemental Table S1.

## Supplementary Materials

The following are available online at https://www.mdpi.com/article/10.3390/cancers13215502/s1, Table S1: TP53 primary mutations and PHANTM combined phenotype score of 107 patients, Table S2: Association of PHANTM combined phenotype score and prognosis: Univariate analysis among three groups.
