# Supplementary material for: TP53 Combined Phenotype Score Is Associated with the Clinical Outcome of TP53-Mutated Myelodysplastic Syndromes
Source: Cancers (Basel). 2021 Nov 2;13(21):5502. doi: 10.3390/cancers13215502 (PMC8582962; doi:10.3390/cancers13215502)
Supplement: Supplementary file 1 [file cancers-13-05502-s001.zip › cancers-1422438-supplementary.pdf]

Supplemental Table S1: TP53 primary mutations and PHANTM combined phenotype score of 107 patients.

TP53 cDNA annotation	PHANTM combined phenotype score
c.154C>T	0.443 ± 0.172
c.637C>T	0.611 ± 0.349
c.814G>A	1.38 ± 0.206
c.742C>T	0.952 ± 0.301
c.524G>A	1.025 ± 0.388
c.646G>A	1.451 ± 0.205
c.633_633delT	1.000
c.826G>C	1.079 ± 0.28
c.536A>T	1.778 ± 0.322
c.1029_1029delA	1.000
c.877-1G>A	1.000
c.659A>G	1.102 ± 0.259
c.427G>A	1.005 ± 0.057
c.536A>G	0.557 ± 0.255
c.755T>C	1.016 ± 0.114
c.722C>G	1.416 ± 0.159
c.470_475delTCCGCG	1.000
c.824G>A	1.49 ± 0.192
c.658T>C	0.986 ± 0.232
c.1037A>G	-0.559 ± 0.149
c.832C>G	1.644 ± 0.296
c.148dupA	1.000
c.517G>A	1.399 ± 0.268
c.742C>G	0.924 ± 0.262
c.743G>A	0.812 ± 0.306
c.817C>T	0.835 ± 0.277
c.383delC	1.000
c.713G>A	1.52 ± 0.195
c.817C>T	0.835 ± 0.277
c.659A>G	1.102 ± 0.259
c.646G>A	1.451 ± 0.205
c.743G>A	0.812 ± 0.306
c.734G>A	0.014 ± 0.675
c.517G>T	0.389 ± 0.258
c.844C>T	1.026 ± 0.283
c.743G>A	0.812 ± 0.306
c.711G>A	0.945 ± 0.237
c.542G>A	0.108 ± 0.399
c.725G>A	1.07 ± 0.39
c.711G>A	0.945 ± 0.237
c.393_395delCAA	1.000
c.273_279delGCCCCTG	1.000

c.380C>T	1.314 ± 0.171
c.376-2A>G	1.000
c.734G>A	0.014 ± 0.675
c.659A>G	1.102 ± 0.259
c.659A>G	1.102 ± 0.259
c.818G>A	1.221 ± 0.111
c.659A>G	1.102 ± 0.259
c.880delG	1.000
c.838A>G	1.097 ± 0.136
c.469G>T	0.304 ± 0.201
c.646G>A	1.451 ± 0.205
c.659A>G	1.102 ± 0.259
c.818G>A	1.221 ± 0.111
c.734G>A	0.014 ± 0.675
c.658T>C	0.986 ± 0.232
c.733G>A	0.772 ± 0.201
c.329G>T	0.253 ± 0.192
c.646G>A	1.451 ± 0.205
c.700T>C	1.071 ± 0.353
c.613dupT	0.731 ± 0.367
c.541C>T	-0.271 ± 0.335
c.641A>G	1.165 ± 0.248
c.701A>G	1.179 ± 0.149
c.713G>A	1.52 ± 0.195
c.309C>G	-0.031 ± 0.11
c.1009C>T	0.300 ± 0.65
c.718A>G	0.738 ± 0.199
c.817C>T	0.835 ± 0.277
c.646G>A	1.451 ± 0.205
c.818G>A	1.221 ± 0.111
c.827C>G	0.677 ± 0.175
c.821T>C	1.142 ± 0.325
c.782+1G>T	1.000
c.672G>A	1.000
c.784G>A	0.018 ± 0.169
c.488A>G	1.341 ± 0.215
c.517G>A	1.399 ± 0.268
c.743G>A	0.812 ± 0.306
c.376-1G>A	1.000
c.713G>A	1.52 ± 0.195
c.818G>A	1.221 ± 0.111
c.427G>A	1.451 ± 0.205
c.579_593delTCTTATCCGAGTGGA	1.000
c.376-1G>A	1.000
c.536A>G	1.102 ± 0.259
c.659A>G	1.102 ± 0.259

c.814G>A	1.38 ± 0.206
c.488A>G	1.341 ± 0.215
c.423C>G	1.129 ± 0.351
c.394A>G	1.044 ± 0.334
c.824G>T	1.241 ± 0.212
c.559+1G>A	1.000
c.140C>G	0.319 ± 0.335
c.533A>C	1.359 ± 0.198
c.193A>T	0.727 ± 0.422
c.1009C>T	0.3 ± 0.65
c.747G>T	0.912 ± 0.538
c.607G>A	0.245 ± 0.37
c.818G>A	1.221 ± 0.111
c.746G>C	1.532 ± 0.256
c.395A>G	1.337 ± 0.069
c.329G>C	1.329 ± 0.128
c.808T>A	1.124 ± 0.272
c.838A>G	1.097 ± 0.136
c.742C>T	0.952 ± 0.301

Supplemental Table S2: Association of PHANTM combined phenotype score and prognosis: Univariate analysis among three groups.

PHANTM combined phenotype score	n	Median OS (95% CI), months	HR (95% CI)	P value
<1	38	19.51 (12.76-41.88)	1	**0.027**
1	16	8.16 (6.74-NA)	1.87 (0.89-3.93)	
>1	53	10.59 (8.98-15.33)	2 (1.19-3.36)	
